# Single-cell profiling reveals pathogenic role and differentiation trajectory of granzyme K^+^CD8^+^ T cells in primary Sjögren’s syndrome

**DOI:** 10.1172/jci.insight.167490

**Published:** 2023-04-24

**Authors:** Ting Xu, Hao-Xian Zhu, Xing You, Jin-Fen Ma, Xin Li, Pan-Yue Luo, Yang Li, Zhe-Xiong Lian, Cai-Yue Gao

**Affiliations:** 1 Department of Rheumatology and Immunology, Guangdong Provincial People’s Hospital (Guangdong Academy of Medical Sciences), Southern Medical University, Guangzhou, Guangdong, China.; 2 School of Medicine, South China University of Technology, Guangzhou, China.; 3 Medical Research Institute, Guangdong Provincial People’s Hospital (Guangdong Academy of Medical Sciences), Southern Medical University, Guangzhou, China.; 4 School of Biomedical Sciences and Engineering, South China University of Technology, Guangzhou International Campus, Guangzhou, China.; 5 Guangdong Provincial People’s Hospital (Guangdong Academy of Medical Sciences), Southern Medical University, Guangzhou, China.

**Keywords:** Autoimmunity, Immunology, Autoimmune diseases, Rheumatology, T cells

## Abstract

Primary Sjögren’s syndrome (pSS) is a systemic autoimmune inflammatory disease mainly defined by T cell–dominated destruction of exocrine glands. Currently, CD8^+^ T cells are thought to be involved in the pathogenesis of pSS. However, the single-cell immune profiling of pSS and molecular signatures of pathogenic CD8^+^ T cells have not been well elucidated. Our multiomics investigation showed that both T cells and B cells, especially CD8^+^ T cells, were undergoing significant clonal expansion in pSS patients. TCR clonality analysis revealed that peripheral blood granzyme K^+^ (GZMK^+^) CXCR6^+^CD8^+^ T cells had higher a proportion of clones shared with CD69^+^CD103^–^CD8^+^ tissue-resident memory T (Trm) cells in labial glands in pSS. CD69^+^CD103^–^CD8^+^ Trm cells featured by high expression of GZMK were more active and cytotoxic in pSS compared with their CD103^+^ counterparts. Peripheral blood GZMK^+^CXCR6^+^CD8^+^ T cells with higher CD122 expression were increased and harbored a gene signature similar to Trm cells in pSS. Consistently, IL-15 was significantly elevated in pSS plasma and showed the capacity to promote differentiation of CD8^+^ T cells into GZMK^+^CXCR6^+^CD8^+^ T cells in a STAT5-dependent manner. In summary, we depicted the immune profile of pSS and further conducted comprehensive bioinformatics analysis and in vitro experimental investigations to characterize the pathogenic role and differentiation trajectory of CD8^+^ Trm cells in pSS.

## Graphical abstract

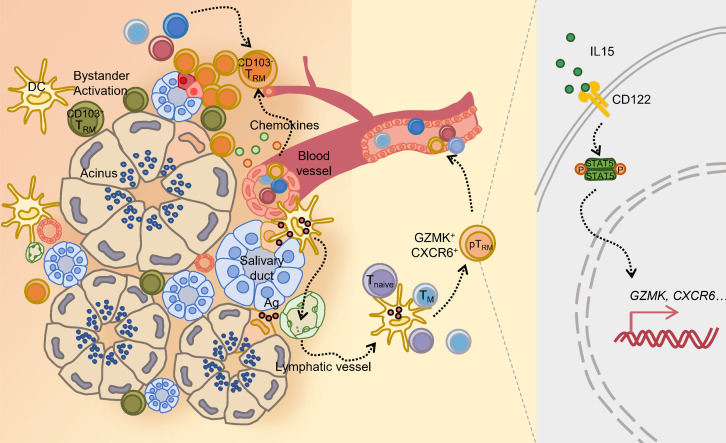

## Introduction

Primary Sjögren’s syndrome (pSS) is one of the most common autoimmune diseases, characterized by immune-mediated progressive injury of exocrine glands such as lacrimal and salivary glands, resulting in xerostomia and xerophthalmia. A relatively high number of pSS patients develop extraglandular manifestations, including renal, pulmonary, and hepatic injury, which may result in significant morbidity and mortality (1). T cells are considered the main contributor to the onset and progression of pSS. However, the immunological and pathogenic properties of pSS are still not well understood (2, 3). Several studies have systematically demonstrated that CD4^+^ T cell subsets, such as Th1, Th17, and T follicular helper (Tfh), are involved in the development of pSS (4, 5). The role of CD8^+^ T cells in pSS has rarely been explored. Recent multiple biological analyses imply that CD8^+^ T cells are related to the severity of pSS (6, 7). Our previous study also confirmed that a large number of CD8^+^ T cells infiltrated the salivary glands of pSS patients and a mouse model, manifested as tissue-resident memory T (Trm) cells and strongly correlated with tissue damage and secretory function loss (8).

CD8^+^ Trm cells coexpressing CD69 and CXCR6 reside in most nonlymphoid tissues, with some in secondary lymphoid organs. They remain positioned within the sites where they initially encountered antigen, providing a rapid response to local antigen restimulation by producing robust inflammatory cytokines, such as IFN-γ and TNF-α (9). Salivary glands are common targets for virus attack and are highly populated with Trm cells, which patrol the salivary gland and protect them from pathogens (10). CD8^+^ Trm cells within a variety of tissue compartments are heterogeneous in phenotype and function, and can be broadly divided into 2 subpopulations based on CD103 expression: CD69^+^CD103^–^CD8^+^ Trm (CD103n CD8^+^ Trm) and CD69^+^CD103^+^CD8^+^ Trm (CD103p CD8^+^ Trm) cells (11). Some tissues, such as intestines and salivary glands, harbor both CD103p and CD103n CD8^+^ Trm subsets, and these 2 populations display distinct functional capabilities in the context of response to infection (12–14). However, the characteristic differences of these Trm subsets and their contributions to pSS remain poorly defined. Single-cell sequencing has been used to understand the heterogeneity, functional plasticity, and differentiation trajectory of different cell types. Our transcriptional analysis showed that CD103p and CD103n CD8^+^ Trm subsets in labial glands (LGs, the salivary glands of the lip belong to the minor salivary glands) displayed unique gene expression profiles. Compared with CD103p CD8^+^ Trm cells, CD103n CD8^+^ Trm cells enhanced the sensitivity to T cell receptor (TCR) stimulation and cytokine production, and were positively correlated with the degree of inflammatory response in pSS.

Recently, several studies have demonstrated that CD8^+^ T cells induced by antigen and specific cytokines, such as TGF-β and IL-15, bear some similar properties to those of Trm cells (15). This implies that individual T cells are actively preconditioned to follow a specific differentiation path already at the naive cell phase (16). However, whether a similar scenario exists in pSS and the signals that control the differentiation and migration of these T cells remain unknown. Our findings revealed a connection between LG hypoproliferative CD8^+^ Trm cells and their peripheral precursors in pSS. We defined a group of CD8^+^ T cells in the periphery marked by GZMK and CXCR6 as the precursors of CD8^+^ Trm cells via single-cell transcription and TCR clonality analysis. Both IL-2 and IL-15 can effectively upregulate CXCR6 expression. Our data further suggested that IL-15 has greater potential to facilitate memory CD8^+^ T cells obtaining a Trm-like signature in a STAT5-dependent manner.

Using single-cell RNA sequencing (scRNA-seq) and single-cell T cell receptor and B cell receptor sequencing (scTCR-seq and scBCR-seq) in peripheral blood and LGs, we illustrate the heterogeneity of the immune cells and the alteration of the immune repertoire in pSS patients. These studies depict the immune profile, identify the potential pathogenic CD8^+^ T cell subsets, and illustrate their differentiation trajectory in pSS.

## Results

### Single-cell transcriptional features of LGs and PBMCs in pSS patients.

**Figure 1:**
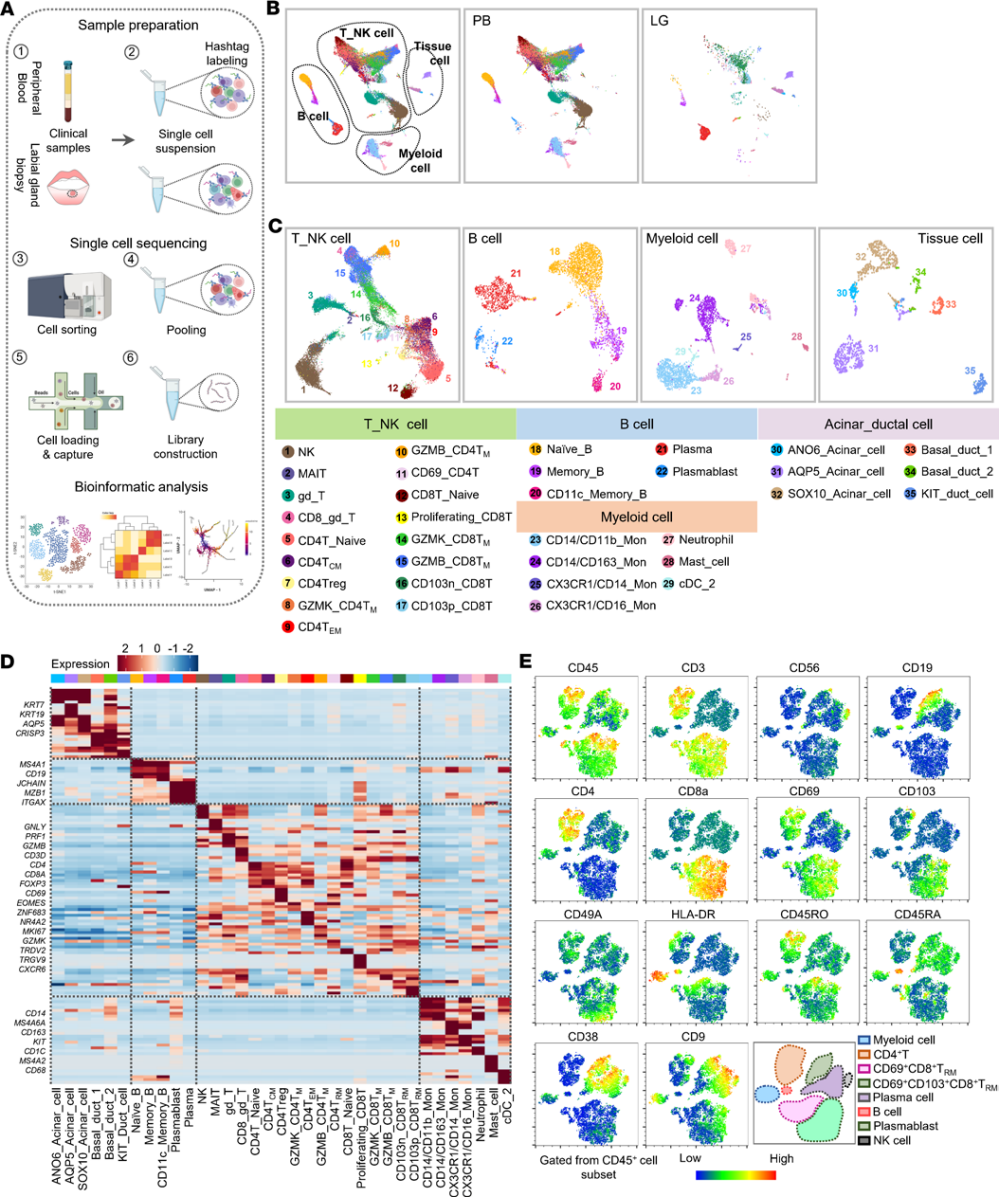
Single-cell transcriptional signatures of LGs and PBMCs in pSS patients. (**A**) Overview of the experimental approach. (**B** and **C**) The UMAP of total cells from LGs and PBMCs, colored by cluster. (**D**) Expression heatmap shows differentially expressed genes of each cluster. Selected genes are indicated to the left, and complete lists of top genes are available in Supplemental Table 5. (**E**) Visualization of LG flow cytometry data by t-SNE. The experiment was performed 3 times.

To determine the cellular composition and gene expression pattern in pSS, we profiled all cells from LGs and peripheral blood in parallel from pSS patients (*n* = 3) and age- and sex-paired healthy controls (HCs) (*n* = 4) by scRNA-seq (Figure 1A, Supplemental Figure 1, A–E, and Supplemental Table 1; supplemental material available online with this article; https://doi.org/10.1172/jci.insight.167490DS1). After removing cells with abnormal gene number and high mitochondrial gene content, we obtained 34,536 cells for further analysis, including 4056 cells derived from LGs and 26,187 from PBMCs. Unbiased clustering identified 4 major clusters, including T and NK cells, B cells, myeloid cells, and tissue cells (Figure 1B). We further classified these major clusters into 35 subclusters according to the top feature genes (Figure 1, C and D, and Supplemental Figure 1, F–J). As previously reported, T cells and B cells are the main infiltrated immune subsets in LGs, and they exhibited tissue-specific transcriptional signatures compared with those in PBMCs (Figure 1, B and C). Dimensional reduction analysis of flow cytometry data with t-distributed stochastic neighbor embedding (t-SNE) showed that the immune component of LGs from pSS patients was similar to that of scRNA-seq (Figure 1E).

### Clonal hyperexpansion of B cells in pSS patients.

We identified 5 B cell subsets based on their genetic characteristics: (a) naive B cells (*TCL1A*); (b) memory B cells (*CD82*, *CXCR4*); (c) CD11c^+^ memory B cells (*TBX21*, *ITGAX*), considered autoimmune-related subsets and precursors of antibody-secreting cells (ASCs) (17, 18); (d) plasmablasts (*CD38*, *MZB1*); and (e) plasma cells (*SDC1*) (Figure 1C and Supplemental Figure 1H). Compared with HC PBMCs, pSS PBMCs had higher proportions of CD11c^+^ memory B cells and plasmablasts (Supplemental Figure 2, A and B). In contrast to peripheral blood, the proportion of plasma cells in LGs of pSS patients was high, and they mainly expressed *IGHA1* and *IGHG1* (Supplemental Figure 2C). Multiplex immunohistochemical staining also confirmed that CD20^–^CD138^+^ cells were colocalized with IgA or IgG in LGs from pSS patients (Supplemental Figure 2D). Moreover, BCR profiling suggested limited diversity and high species abundance in B cell clonal expansion in LGs from pSS patients (Supplemental Figure 2, E and F). Taken together, our data suggest a vigorous B cell response in pSS.

### Clonal expansion of CD8^+^ T cells in pSS patients.

**Figure 2:**
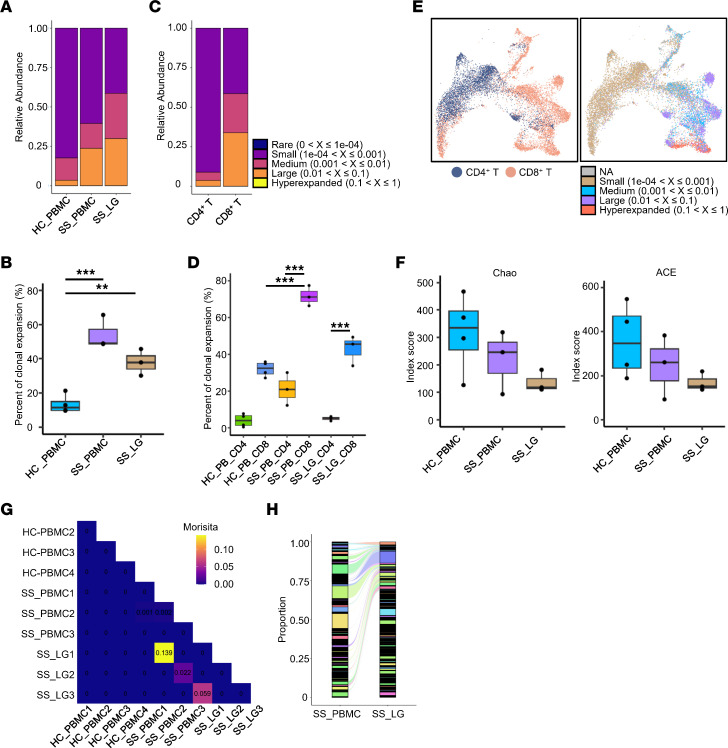
Clonal expansion of CD8^+^ T cells in pSS patients. (**A**) Clone size of PBMCs and LG T cells from HCs and pSS patients. (**B**) Clonal expansion percentage (at least 2 or more cells were included in each clonotype) of PBMCs and LG memory T cells in HCs and pSS patients. (**C**) Bar graph shows clonal size of CD4^+^ or CD8^+^ T cells in pSS patients. (**D**) Clonal expansion percentages of all CD4^+^ or CD8^+^ memory T cells from HCs and pSS patients. (**E**) UMAP plot shows the clonal distribution of CD4^+^ T cells and CD8^+^ T cells of pSS patients. (**F**) The Chao and ACE index scores for CD8^+^ T cells in HCs and pSS patients. (**G**) Morisita index of shared clones in CD8^+^ T cells of PBMCs and LGs from the same patient. (**H**) Alluvial plot shows the shared clones in CD8^+^ T cells of LGs and PBMCs from pSS patients. In the box-and-whisker plots in **B**, **D**, and **F**, each data point represents 1 individual, horizontal lines indicate medians, bounds of the boxes represent IQRs, and whiskers extend to the farthest data points. **P < 0.01; ***P < 0.001 by 1-way ANOVA with Dunnett’s multiple-comparison test (**B** and **D**).

Since T cells play a critical pathogenic role in pSS, we assessed T cell expansion by scTCR-seq. Consistent with previous research (19), both pSS PBMCs and LGs had a higher percentage of T cell clonal expansion compared with HC PBMCs, indicating that T cells in pSS experienced a more active immune response (Figure 2, A and B). We compared the differences between CD4^+^ and CD8^+^ T cell clonal expansion. The expanded T cells were mainly CD8^+^ T cells in pSS, which had higher clonal amplification rates and larger single clones (Figure 2, C–E). These data indicated that the difference in TCR clonal expansion rate between HC and pSS patients was mainly contributed by CD8^+^ T cells. Consistently, we observed a significant reduction in the clonotype species diversity of CD8^+^ T cells in pSS according to Chao and ACE scoring (Figure 2F). We next examined whether shared clones existed across patients and between LGs and PBMCs. Although each patient had a unique TCR repertoire, CD8^+^ T cells from peripheral blood and LGs in pSS patients had shared clones, suggesting that LG CD8^+^ T cells may be derived from the periphery (Figure 2, G and H, and Supplemental Figure 3). Our previous work has demonstrated an important pathogenic role for CD8^+^ T cells in a pSS mouse model (8). In the present study, the overall TCR analysis further hinted that CD8^+^ T cells undergo robust clonal expansion in pSS, and might also play a critical role in the onset and progress of pSS.

### CD103n CD8^+^ Trm cells are more cytotoxic than CD103p CD8^+^ Trm cells.

**Figure 3:**
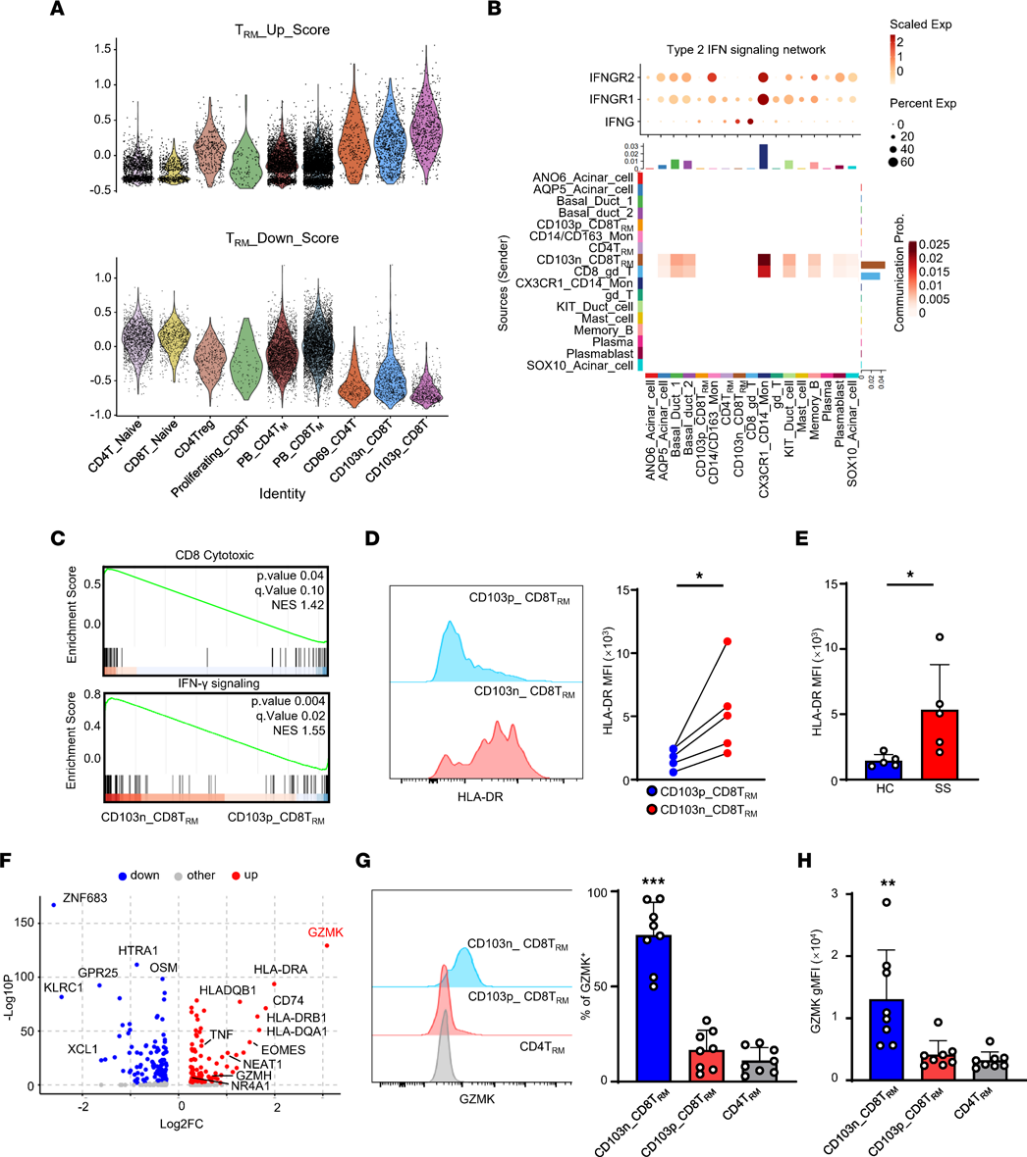
CD103^–^CD8^+^ Trm cells are more cytotoxic than CD103^+^CD8^+^ Trm cells. (**A**) Violin plots displaying Trm_Up or Trm_Down scores of each T cell cluster. PB_CD4T_M_ includes CD4^+^ Tcm, GZMB^+^CD4^+^ Tm, and GZMK^+^CD4^+^ Tm. PB_CD8T_M_ includes GZMB^+^CD8^+^ Tm and GZMK^+^CD8^+^ Tm. CD103n_CD8T refers to CD103^–^CD8^+^ T cells. CD103p_CD8T refers to CD103^+^CD8^+^ T cells. (**B**) CellChat-derived the type 2 IFN signaling network among major cell types in LGs from pSS patients. Top: The shade level of the bar represents the scaled expression of genes, and the circle size represents the percentage of cells expressing the genes. Bottom: CellChat scoring according to *IFNG* and IFN-γ receptor expression level. (**C**) The GSEA plot shows the enrichment score of CD8^+^ cytotoxic and IFN-γ signaling pathways between CD103^–^CD8^+^ Trm and CD103^+^CD8^+^ Trm cells. *P* values are shown on the right of the plot. (**D**) Flow cytometry results (left) and statistical analysis (right) of HLA-DR expression level of CD8^+^ Trm subpopulations in LGs of pSS patients (*n* = 5). The blue dots represent CD103^+^CD8^+^ Trm and red CD103^–^CD8^+^ Trm cells. (**E**) Statistical analysis compares the difference in expression level of HLA-DR in CD103^–^CD8^+^ Trm cells between pSS patients (*n* = 5, red bar) and HCs (*n* = 5, blue bar). (**F**) Volcano plot shows the differentially expressed genes between CD103^–^CD8^+^ Trm and CD103^+^CD8^+^ Trm cells. (**G**) Flow cytometry result (left) and statistical analysis (right) of the expression level of GZMK in different LG Trm cells of pSS patients. (**H**) Statistical analysis comparing the difference in expression level of GZMK in different LG Trm cells of pSS patients. Data are presented as mean ± SD. *P < 0.05; **P < 0.01; ***P < 0.001 by 2-tailed, paired Student’s *t* test (**D**), 2-tailed, unpaired Student’s *t* test (**E**), or 1-way ANOVA with Dunnett’s multiple-comparison test (**G** and **H**).

It has been shown that CD8^+^ Trm cells in salivary glands contain 2 subsets, CD103^–^ and CD103^+^, both of which express CD69 at a moderate level (10). Considering that CD8^+^ T cells in pSS LGs, including CD103n CD8^+^ T and CD103p CD8^+^ T cells, had a high score in the Trm signature gene set, but the lowest score in Trm downregulated genes, we defined them as Trm cells (Figure 3A and Supplemental Figure 4A). IFN-γ is known to be involved in the pathogenesis of pSS (20). Our signaling network analysis revealed that CD103n CD8^+^ Trm cells expressed higher levels of *IFNG* and interacted with cells expressing high levels of the IFN-γ receptor, including tissue cells, monocytes, and B cells (Figure 3B). Next, we performed gene set enrichment analysis (GSEA) to compare the difference in gene expression profiles. Our data showed that CD8^+^ cytotoxic signatures and IFN-γ signaling–related genes were significantly enriched in CD103n CD8^+^ Trm cells (Figure 3C). Compared with CD103p CD8^+^ Trm cells, CD103n CD8^+^ Trm cells in pSS LGs expressed higher levels of HLA-DR (Figure 3D). Our results indicated that CD103n CD8^+^ Trm cells were more cytotoxic and active than their CD103^+^ counterpart. Furthermore, as compared with HCs, CD103n CD8^+^ Trm cells in pSS also expressed higher levels of HLA-DR, suggesting CD103n CD8^+^ Trm cells might be a disease-associated activated subset (Figure 3E). Previous research has found that HLA-DR^+^CD8^+^ T cells are related to various disease signatures in pSS, including focus score and presentation of autoantibodies (7). Our differential expression analysis determined that *GZMK* was the most representative gene of the CD103n CD8^+^ Trm subset (Figure 3F). And flow cytometry intracellular labeling confirmed that CD103n CD8^+^ Trm cells were the main source of GZMK (Figure 3, G and H). Moreover, CD103n CD8^+^ Trm cells coexpressed GZMB and GZMK (Supplemental Figure 4B). In alignment with the latest published data (21), our results suggested that CD103n CD8^+^ Trm cells were clonally expanded and the major cytokine producers in pSS.

### Pathogenic role of CD103n CD8^+^ Trm cells in pSS.

**Figure 4:**
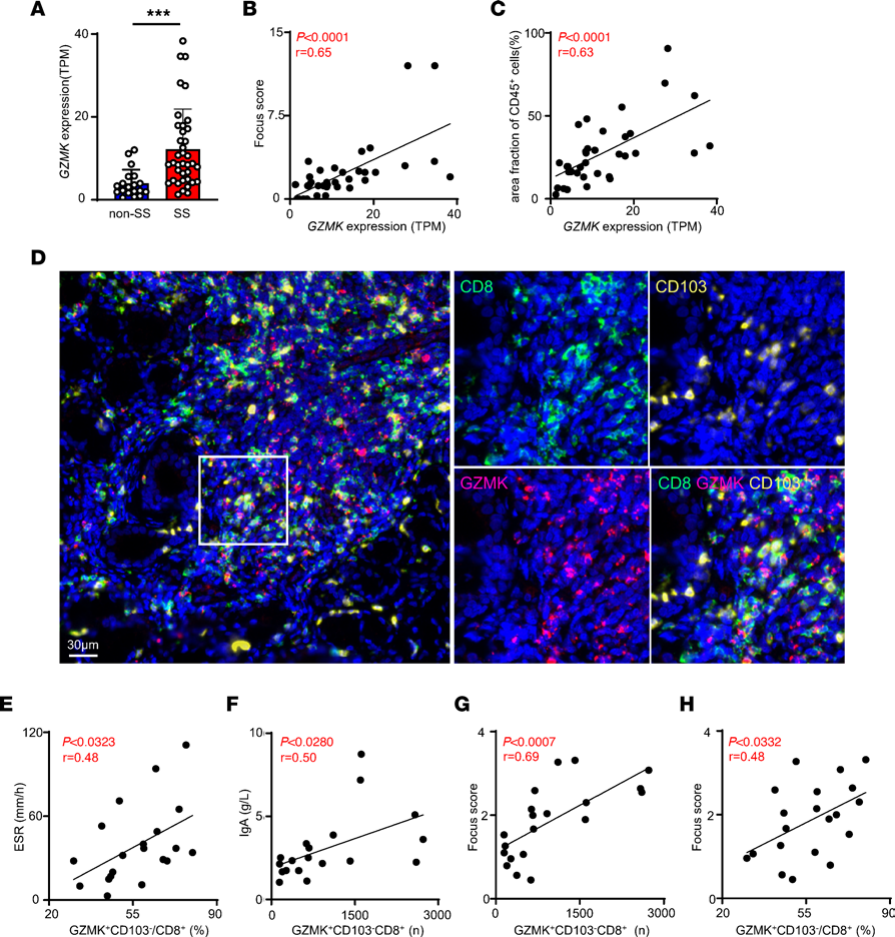
Pathogenic role of GZMK^+^CD8^+^ T cells in pSS. (**A**) The *GZMK* gene expression level in LGs of pSS patients and non-SS patients with sicca. (**B** and **C**) Correlation between the expression level of *GZMK* with focus score and the percentage area fraction of CD45^+^ cells. For **A**–**C**, the TPM value of *GZMK* was transformed from GEO GSE173808 microarray count data. (**D**) Representative multiplex staining of CD8 (green), CD103 (yellow), and GZMK (red) in LGs from pSS patient. Scale bar: 30 μm. The experiment was performed 2 times. (**E**) Correlation between GZMK^+^CD103^–^CD8^+^ T cells and erythrocyte sedimentation rate (ESR) in **D** (*n* = 20). (**F**) Correlation between GZMK^+^CD103^–^CD8^+^ T cells and the level of serum IgA in **D** (*n* = 20). (**G** and **H**) Correlation between the number or frequency of GZMK^+^CD103^–^CD8^+^ T cells and focus score of LGs from pSS patients in **D** (*n* = 20). Data are presented as mean ± SD. ***P < 0.001 by 2-tailed, unpaired Student’s *t* test (**A**). *P* values in **B**, **C**, and **E**–**H** were derived using Pearson’s correlation.

As CD103n CD8^+^ Trm cells were the main source of GZMK, we used GZMK as the surrogate marker for CD103n CD8^+^ Trm cells in LGs. To further investigate the role of CD103n CD8^+^ Trm cells in pSS, we analyzed a publicly available LG bulk RNA-seq data set containing 38 pSS patients and 19 non-SS patients with sicca (22). We found that the expression of GZMK was significantly upregulated in pSS LGs and positively related to patients’ focus score and area fraction of CD45^+^ cells, indicating that CD103n CD8^+^ Trm cells play a pathogenic role in pSS (Figure 4, A–C). Our multiplex immunohistochemistry staining further confirmed that the vast majority of CD103^–^CD8^+^ cells in pSS LGs were GZMK^+^ cells (Figure 4D). Then, we analyzed the relationship between the infiltration of GZMK^+^CD103^–^CD8^+^ Trm cells in LGs and the clinical and histological indices of pSS. The infiltration of GZMK^+^CD103^–^ Trm cells was positively related to erythrocyte sedimentation rate and surprisingly, the serum IgA but not IgG level (Figure 4, E and F, and Supplemental Figure 5C). In addition, both the frequency and cell number of GZMK^+^CD103^–^CD8^+^ Trm cells were positively related to focus score (Figure 4, G and H). However, CD103p CD8^+^ Trm cells showed no correlation with the focus score (Supplemental Figure 5, A and B). Our data also showed the colocalization of CD8^+^ cells and CD20^+^Ki67^+^ cells, indicating that GZMK^+^CD103^–^CD8^+^ Trm cells might promote the antibody secretion of IgA^+^ B cells in LGs of pSS (Supplemental Figure 5E). Conclusively, our data showed that CD103n CD8^+^ Trm cells, but not CD103p CD8^+^ Trm cells, were the predominant pathogenic subset in pSS.

### GZMK^+^CXCR6^+^CD8^+^ T cells are Trm precursors.

**Figure 5:**
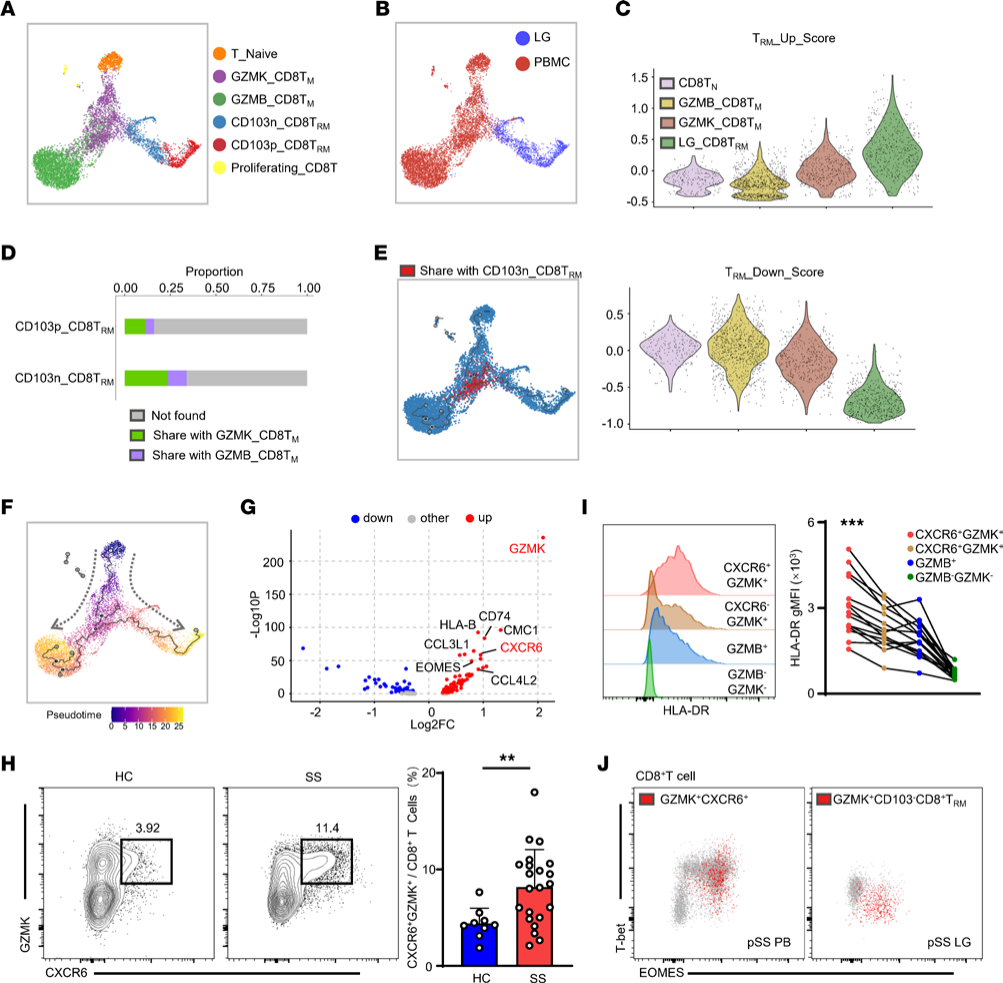
GZMK^+^CXCR6^+^CD8^+^ T cells are Trm precursors. (**A**) UMAP plot shows cluster distribution of CD8^+^ T cells in LGs and PBMCs from pSS patients. (**B**) UMAP plot shows the tissue distribution of CD8^+^ T cells. (**C**) Trm score of the PBMC CD8^+^ T cell cluster and LG Trm cells. LG_CD8T_RM_ includes CD103^+^CD8^+^ Trm and CD103^–^CD8^+^ Trm cells. (**D**) Proportion of clone types shared with CD103^+^CD8^+^ Trm or CD103^–^CD8^+^ Trm cells. (**E**) The distribution of clones shared with CD103^–^CD8^+^ Trm cells in GZMK_CD8T_M_. (**F**) Monocle3-derived pseudotime and trajectory line of CD8^+^ T cells on UMAP plot. (**G**) Volcano plot shows the differentially expressed genes in cells with shared clones of GZMK_CD8T_M_. (**H**) Frequency of CXCR6^+^GZMK^+^CD8^+^ T cells in PBMC CD8^+^ T cells from pSS patients (*n* = 22) and HCs (*n* = 9). Blue bar represents cells from HCs and red pSS patients. (**I**) The geometric mean fluorescence intensity of HLA-DR in CXCR6^+^GZMK^+^CD8^+^ T cells, CXCR6^–^GZMK^+^CD8^+^ T cells, GZMB^+^CD8^+^ T cells, and GZMB^–^GZMK^–^CD8^+^ T cells in PBMCs from pSS patients (*n* = 15). Red dots represent GZMK^+^CXCR6^+^CD8^+^ T cells and blue GZMB^+^CD8^+^ T cells. (**J**) Representative flow cytometry plots show the EOMES expression of CXCR6^+^GZMK^+^CD8^+^ T cells and GZMK^+^CD103^–^CD8^+^ Trm cells. The experiment was performed 3 times. Data are presented as mean ± SD. **P < 0.01; ***P < 0.001 by 2-tailed, unpaired Student’s *t* test (**H**) or 1-way ANOVA with Dunnett’s multiple-comparison test (**I**).

To gain further insight into the lineage commitment of pSS CD8^+^ T cells, we clustered CD8^+^ T cells from both LGs and PBMCs and identified 6 cell groups, including naive, GZMK^+^ memory, GZMB^+^ memory, and proliferating CD8^+^ T cells in PBMCs and 2 groups of Trm cells in LGs (Figure 5, A and B, Supplemental Figure 6, A–C). As previously described, we found a population of CD8^+^ T cells in PBMCs that shared the same CDR3 amino acid sequences with LG CD8^+^ T cells (Figure 2, G and H). We also found that GZMK^+^ memory T (Tm) cells in PBMCs exhibited Trm characteristics at the transcriptional level, implying their potential for differentiation into Trm cells (Figure 5C). Compared with CD103p CD8^+^ Trm cells, CD103n CD8^+^ Trm cells had higher proportions of clones shared with peripheral CD8^+^ T cells, which were mostly GZMK^+^ Tm cells (Figure 5D and Supplemental Figure 6, D and E). We further found that the clones shared with LG CD103n CD8^+^ Trm cells were mainly distributed in GZMK^+^ Tm cells (Figure 5E). Making use of Monocle3, we reconstructed the developmental trajectory on the dimensionality reduction of CD8^+^ T cells (Figure 5F). Naive T cells went through GZMK^+^ Tm and terminally differentiated into GZMB^+^ Tm or LG Trm cells. GZMK^+^ Tm cells were located at the crossroad of differentiation trajectories, where shared clones were distributed. We also confirmed that GZMK^+^ Tm cells possessed an intermediate phenotype and potentially turned into CD103n CD8^+^ Trm cell in LGs (Figure 5F). Consistently, clonally shared GZMK^+^ Tm cells exhibited higher *GZMK* and *CXCR6* transcription compared with other CD8^+^ T cells in the periphery (Figure 5G). To further detect these clone-sharing cells by flow cytometry, we identified GZMK and CXCR6 as markers. Indeed, the proportion of CXCR6^+^GZMK^+^CD8^+^ T cells in the peripheral blood of pSS patients was significantly higher than that of HCs (Figure 5H). Moreover, CXCR6^+^GZMK^+^CD8^+^ T cells in pSS patients expressed higher HLA-DR, indicating they were an activated phenotype (Figure 5I). Both CXCR6^+^GZMK^+^CD8^+^ T cells in the periphery and CD103n CD8^+^ Trm cells in LGs expressed the T-box transcription factor eomesodermin (EOMES) (Figure 5J). Indeed, we found that overexpression of EOMES in CD8^+^ T cells was capable of upregulating the expression of GZMK by analyzing published transcriptome sequencing data (Supplemental Figure 6F) (23). These findings suggested that peripheral CXCR6^+^GZMK^+^CD8^+^ T cells might be a subset associated with disease progression, whose phenotype and function might be orchestrated by EOMES.

### IL-15 primes CD8^+^ T cells to differentiate into Trm precursors in pSS.

**Figure 6:**
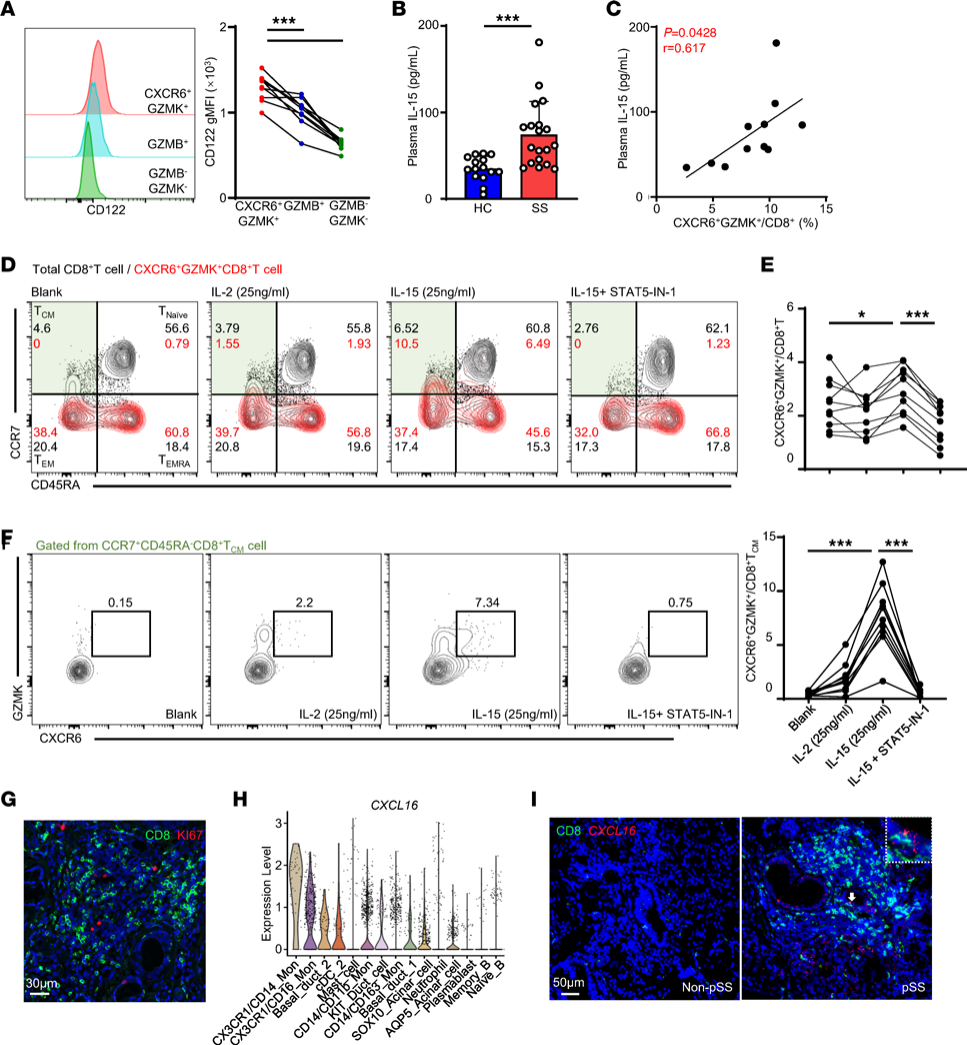
IL-15 primes CD8^+^ T cells to differentiate into Trm precursors in pSS. (**A**) The geometric mean fluorescence intensity of CD122 in CXCR6^+^GZMK^+^CD8^+^ T cells, GZMB^+^CD8^+^ T cells, and GZMB^–^GZMK^–^CD8^+^ T cells in PBMCs from pSS patients (*n* = 10). Red dots represent GZMK^+^CXCR6^+^CD8^+^ T cells, blue GZMB^+^CD8^+^ T cells, and green GZMB^–^GZMK^–^CD8^+^ T cells. (**B**) IL-15 concentration in blood plasma from pSS patients (*n* = 19) and HCs (*n* = 15). Blue bar represents cells from HCs and red pSS patients. (**C**) Correlation between frequency of CXCR6^+^GZMK^+^CD8^+^ T cells and blood plasma IL-15 concentration in pSS patients with paired PBMCs and blood plasma data. (**D**) Representative flow cytometry plots show the CD45RA and CCR7 expression of CXCR6^+^GZMK^+^CD8^+^ T cells after cytokine treatment. (**E**) Frequency of CXCR6^+^GZMK^+^ cells among CD8^+^ T cells after treating with IL-2, IL-15, and STAT5 inhibitor (STAT5-IN-1) for 48 hours. (**F**) Flow cytometry result (left) and statistical analysis (right) shows the frequency of CXCR6^+^GZMK^+^ cells among CD8^+^ Tcm cells after treating with IL-2, IL-15, and STAT5 inhibitor (STAT5-IN-1) for 48 hours. (**G**) Representative multiplex immunohistochemical staining of CD8 (green) and Ki67 (red) in LGs of pSS patients. Scale bar: 30 μm. The experiment was performed 3 times. (**H**) Violin plot showing the gene *CXCL16* expression level in the main clusters of LGs from pSS patients. (**I**) Representative images of *CXCL16* RNAscope in situ hybridization (red) and CD8 staining (green). The experiment was performed 2 times. Scale bar: 50 μm. Data are presented as mean ± SD. *P < 0.05; ***P < 0.001 by 1-way ANOVA with Dunnett’s multiple-comparison test (**A**, **E**, and **F**), 2-tailed, unpaired Student’s *t* test (**B**), or Pearson’s correlation (**C**).

Published work has demonstrated that IL-15 is capable of effectively upregulating CXCR6 expression (24). In this study, we surprisingly found that the expression level of CD122 in peripheral CXCR6^+^GZMK^+^CD8^+^ T cells was higher than that in GZMB^+^CD8^+^ T cells and GZMB^–^GZMK^–^CD8^+^ T cells (Figure 6A). Moreover, the plasma IL-15 level was elevated and positively correlated with the proportion of peripheral CXCR6^+^GZMK^+^CD8^+^ T cells in pSS patients (Figure 6, B and C). This is consistent with published data showing that IL-15 may contribute to CXCR6^+^CD8^+^ T cell formation in vivo (24). Our data further demonstrated that IL-15 was capable of upregulating the proportion of CXCR6- and GZMK-coexpressing populations in human CD8^+^ T cells in vitro, suggesting that IL-15 plays an important role in increasing and sustaining peripheral CXCR6^+^GZMK^+^CD8^+^ T cells in pSS patients (Figure 6, D and E). Of note, compared with IL-2, which was also reported as a CXCR6 inducer, IL-15 had a stronger ability to induce the expression of CXCR6 in the CD8^+^ T central memory (Tcm) subset (Figure 6, D and F). IL-15–induced JAK/STAT signaling along with the PI3K/AKT/mTOR and MAPK pathways is fundamentally important for memory T cell formation, activation, homeostasis, and survival (25, 26). Here, we found that inhibition of STAT5 in CD8^+^ T cells blocked the IL-15–induced expression of CXCR6 and GZMK (Figure 6, E and F). We concluded that STAT5 plays a key role in the formation of IL-15–dependent Trm precursors in pSS. As evidenced by the rare expression of proliferation marker Ki67 in LG CD8^+^ T cells (Figure 6G), CD103n CD8^+^ Trm cells may mainly have originated from PBMCs, rather from in situ expansion. CXCL16, the CXCR6 ligand, plays a critical role in Trm cell trafficking and residence. We found that it was expressed by several different kinds of cells in LGs (Figure 6H). Meanwhile, *CXCL16* was detected in CD8^+^ T cell enrichment areas using RNA in situ hybridization and its transcription level was increased in pSS (Figure 6I and Supplemental Figure 6G). These results indicated that a CXCR6/CXCL16 axis might be involved in the infiltration and colonization of CXCR6^+^GZMK^+^CD8^+^ T cells in LGs, which is consistent with a very recent report that also showed, utilizing a cell-cell communication algorithm, that CXCL16-secreting cells signal to clonal CD8^+^ T cells via CXCL16/CXCR6 in cerebrospinal fluid from a cognitively impaired patient to promote their aggregation at the lesion site (27). Thus, our results suggest that disease-associated CD8^+^ Trm cells in LGs originated from CD122-expressing CD8^+^ T cells.

## Discussion

pSS is characterized by an abnormal immune response in exocrine glands. Several studies have observed an enriched immune infiltration in exocrine glands (mainly salivary glands) in pSS patients via multiplexed immunohistochemical staining, including (but not limited to) T cells, B cells, and myeloid cells (28). Immune infiltration was the major contributor of tissue damage and secretory function loss in pSS. The tissue cells in pSS can also exacerbate the immune response and promote disease progression by upregulating chemokines and adhesion molecules to increase immune cell recruitment and immune synapse maintenance (5). However, the local immune niche within salivary glands and the lineage development of pathogenic immune cells in pSS are not well understood. In the present study, we comprehensively describe the immune composition and their transcriptional features in salivary glands of pSS patients, and most importantly, we report the cytotoxic role of GZMK^+^CD8^+^ T cells in pSS and their differentiation potency.

Ectopic germinal center (GC) structures have been frequently observed in salivary glands along with the disease development, suggesting the presence of vigorous lymphocyte activation and clonal expansion in pSS patients (29). Here we found a reduction in TCR and BCR diversity in pSS patients compared with age- and sex-matched HCs, and this result also suggested the possibility of a broader antigen-driven immune response. Importantly, the ectopic B cell zone with great proliferation potential based on Ki67 expression was surrounded by a large quantity of ASCs in salivary glands of pSS patients. GC B cells underwent autoantigen-based BCR affinity selection and class switch recombination, eventually differentiating into autoreactive ASCs in situ. Recent studies have reported the enrichment of CD11c^+^T-bet^+^ B cells in autoimmune diseases and their association with autoantibody production (17, 18). In the present study, we found CD11c^+^T-bet^+^ B cells also increased specifically in the periphery of pSS patients. Taken together, our findings provide a theoretical basis and clues for further exploring autoreactive clonotypes.

Our published work has described the remission of salivary gland pathology and recovery of salivary secretion in SS mouse models when CD8^+^ T cells were absent, suggesting a dominant role for CD8^+^ T cells in the pathogenesis of SS (8). In this study, CD8^+^ T cells within LGs of pSS patients rarely proliferated in situ throughout the course of disease, but were prone to be recruited and differentiated from the periphery, and exhibited a Trm phenotype, mainly including CD103n CD8^+^ Trm and CD103p CD8^+^ Trm subsets. It has been shown that CD103n CD8^+^ Trm cells can be derived from CXCR3-dependent recruiting cells within inflamed areas and play a critical role in controlling infection (30). We found that after transfer into recipient mice, CD69^–^CD8^+^ T cells were also capable of differentiating into CD103n Trm cells in salivary glands in a short period, but fewer of them transformed into the CD103^+^ subset (data not shown). In this report, we further describe an increased percentage of CD103p CD8^+^ Trm cells in LGs and a tendency to disperse in lymphocyte-infiltrated foci as the disease progresses (Supplemental Figure 5D). These results suggest that the transformation of peripheral pathogenic CD8^+^ T cells into CD103p CD8^+^ Trm cells in salivary glands of pSS patients might undergo a longer period of antigen and cytokine stimulation in comparison with their CD103^–^ counterparts. Therefore, the majority of CD103p CD8^+^ Trm cells in salivary glands of pSS patients might be not the disease-related T cells at the not-very-late stage of disease. This might be the reason why CD103p CD8^+^ Trm cells showed lower cytotoxicity and a smaller proportion of clonotype sharing with peripheral cells compared with their CD103^–^ counterparts.

Published work has demonstrated that HLA-DR^+^CD8^+^ T cells in LGs of pSS patients were positively correlated with disease severity and autoantibody level (6). Here we found that the HLA-DR^+^CD8^+^ T cells were mainly GZMK^hi^ CD103n CD8^+^ Trm cells. GZMK^+^CD8^+^ T cells were originally known as age-associated T cells. However, recent studies have reported their various functions in autoimmune disease and tumorous diseases (31). More recent work showed that GZMK^+^CD8^+^ T cells enriched in human inflamed tissue were the major inflammatory cytokine producer and showed the potential to drive inflammation (21). We found that GZMK^+^CD8^+^ Trm cells in LGs of pSS patients also had a high level of *IFNG* and *TNF* transcription. In addition to acting as an inducer in target cell apoptosis, IFN-γ signaling also plays a role in the regulation of the GC by activating B cell–intrinsic IFN-γ receptor signaling pathways (32). This might explain the correlation between serum IgA levels and GZMK^+^CD8^+^ Trm cells. Moreover, GZMK may induce secretion of proinflammatory cytokines or induce cell death directly (21). For example, extracellular GZMK promotes a proinflammatory response in endothelial cells and innate immune cells (33, 34). GZMK also significantly enhances senescence-associated secretory phenotype components either alone or in combination with IFN-γ (31). Taken together, these results show that GZMK^+^CD8^+^ T cells might play a critical pathogenic role in pSS.

To adapt to the destination-tissue microenvironment, the peripheral T cells may upregulate certain residency molecules and alter the metabolic program. In contrast, the CDR3 region of the TCR remains stable once T cells mature from the thymus. Therefore, based on TCR sequence alignment, we found significant clonal sharing between peripheral GZMK^+^CD8^+^ T cells and LG CD8^+^ Trm cells, suggesting that peripheral GZMK^+^CD8^+^ T cells have the potential to differentiate into CD8^+^ Trm cells after being trained by the LG environment. Moreover, peripheral GZMK^+^CD8^+^ T cells displayed pre-Trm transcriptional profiles. CD103n CD8^+^ Trm cells in LGs also expressed high levels of GZMK both at the mRNA and protein level. Antigen-experienced dendritic cells migrate from tissues to secondary lymphoid organs, relying on chemokine receptor expression such as CCR7. Antigen-specific CD8^+^ T cells are primed to form pre-CD8^+^ Trm cells upon TCR stimulation and certain cytokine environments (35, 36). In this study, our data suggest that CXCR6^+^GZMK^+^CD8^+^ T cells in blood may represent Trm precursors. Additional studies are still required to investigate this possibility. IL-15 signaling can induce CXCR6 and GZMK expression on memory CD8^+^ T cells. Taken together, our results show a potential pathogenic role and the differentiation trajectory of GZMK^+^CD8^+^ T cells in pSS. However, the specific pathogenic mechanism of GZMK^+^CD8^+^ T cells in pSS needs to be further explored.

## Methods

### Clinical sample collection.

Fifty-five pSS patients were recruited by the Rheumatology and Immunology Department of Guangdong Provincial People’s Hospital from March 2020 to October 2022. All patients met the 2002 American-European Consensus Group criteria (37) and did not receive any glucocorticoids or immunosuppressants 3 months prior to sampling; individuals with cancer, infection, or other autoimmune diseases were excluded from the study. Twenty-nine individuals who were admitted to the hospital with dry mouth or dry eyes but were undiagnosed for any disease were recruited as HCs. Blood samples were obtained from pSS patients and 29 HCs. Blood samples were used in scRNA-seq, PBMC phenotyping, and blood plasma IL-15 ELISAs. LG tissue biopsy specimens were obtained from pSS patients and 5 individuals who suffered from dry mouth but no focal lymphocytic infiltration. Clinical data were obtained at the time of sampling. Details of all participants are shown in Supplemental Table 2.

### LG cell and PBMC isolation.

The method for isolation of LG cells was previously described (38), the isolation of PBMCs was accomplished by density gradient centrifugation. First, the plasma was removed after peripheral blood was centrifuged at 450*g* for 5 minutes at room temperature. Next, we diluted the blood cells with an equal volume of saline. Then, the cell diluent was layered on the top of Ficoll solution (Lymphoprep, STEMCELL Technologies) and was centrifuged at 800*g* for 20 minutes (acceleration 6, brake 2). Finally, the mononuclear cells at the interface were carefully harvested.

### Single-cell library generation.

Once the LB cells were isolated, we sorted CD45^+^ cells and CD326^+^ tissue cells by BD FACSAria II. First, the cells were resuspended by cell staining buffer (BioLegend, 420201) and blocked with Human TruStain FcX Fc Blocking reagent (BioLegend) at 4°C for 10 minutes. Then, the cells were labeled for CD45, CD326 (only for LG cells), and with TotalSeq-C Hashtags (BioLegend) at 4°C for 30 minutes. After washing with cell staining buffer at 4°C for 5 minutes (3 times), the cells were resuspended in Dulbecco’s PBS (Sangon Biotech, E607009-0600). Finally, the LG cells and PBMCs from the same patient were mixed up to a total of 30,000 cells at 1300 cells/μL. Single-cell libraries were generated following the 10× Genomics protocol using Chromium Next GEM Single Cell 5′ Reagent Kits v2 (dual index).

### Single-cell data processing and quality control.

The sequencing data were processed by CellRanger (version 6.1.2; https://github.com/10XGenomics/cellranger). CellRanger multi was run with default parameters using raw data for each sample, mapping on the human genome (GRCh38/hg38) using STAR (version 2.7.10a; https://github.com/alexdobin/STAR). The reference genome is refdata-gex-GRCh38-2020-A for gene expression, and the refdata-cellranger-vdj-GRCh38-alts-ensembl-5.0.0 for VDJ. Seurat (version 4.1.0; https://github.com/satijalab/seurat) was used for sequential analyses. The preprocessed data matrix was imported by Read10X and transformed to a Seurat object using the CreateSeuratObject function for each sample. The mixed sample was split by HTODemux or the barcode to the sampletag file output from Loupe Browser 6 with AddMetaData. Log-normalization was performed using NormalizeData, and the top 2000 most variable genes were identified by the ‘‘vst’’ method in FindVariableFeatures. Finally, ScaleData, RunPCA, FindNeighbors, FindClusters, RunTSNE, and RunUMAP were used for downstream analyses. The doublets were removed by DoubletFinder (version 2.0; https://github.com/chris-mcginnis-ucsf/DoubletFinder). We removed cells with more than 6000 or less than 200 unique genes or more than 25% mitochondrial genes. Finally, we obtained 34,536 cells for analysis, including 4056 cells derived from LGs and 26,187 from PBMCs. To correct for batch effects, we applied canonical correlation analysis to integrate each sample. Briefly, FindIntegrationAnchors (dims from 1 to 40) and IntegrateData (dims from 1 to 30) were used for identifying anchors and creating an integrated expression matrix for all cells with default parameters. Principal component analysis (PCA) was used for dimensionality reduction using the top 2000 highly variable genes. Using the first 30 principal components and the resolution setting to 0.6 as input, the data were visualized in 2 dimensions with Uniform Manifold Approximation and Projection (UMAP). Dimplot and Featureplot were used for visualizing the dimensionality reduction and distribution of genes.

### GSEA.

To reduce the effects of noise and outliers, we created a supercell matrix by calculating the mean normalized expression value of each gene in every 50 randomly selected cells from CD103n CD8^+^ Trm (total 18 supercells) or CD103p CD8^+^ Trm (total 13 supercells) clusters. The differences in gene expression between the 2 clusters were calculated using LIMMA package (version 3.44.3; https://git.bioconductor.org/packages/limma). GSEA was performed using ClusterProfiler package (version 3.16.1; https://github.com/YuLab-SMU/clusterProfiler). Preranked genes were used in GSEA for calculating *P* values and enrichment scores. The gene sets used in this study are listed in Supplemental Table 3. The “IFN-γ signaling” gene set was from the GSEA database (https://github.com/GSEA-MSigDB/gsea-desktop) and the “CD8 cytotoxic” gene list was obtained from published literature (39).

### Differential gene expression analysis.

Differentially expressed genes shared between clonal GZMK^+^CD8^+^ Tm cells and other PBMCs were identified using the FindMarkers function from Seurat using Wilcoxon’s test, Bonferroni’s *P*-value correction, and 0.25 as the log(fold change) threshold. Significantly differentially expressed genes with a *P* value of less than 0.01 are labeled red (increased) or blue (decreased).

As for microarray data analysis, we downloaded the LG tissue transcriptome of SS patients (GSE173808) from the NCBI Gene Expression Omnibus (GEO) database and performed gene annotation to transform the probe names to symbols; the expression matrix was then log_2_-normalized and transformed to TPM. The normalized matrix was analyzed for *GZMK* gene expression using Student’s *t* test and the correlation between *GZMK* gene expression and focus score or area fraction of CD45^+^ cells was analyzed using Pearson’s correlation with GraphPad Prism 8.

### Trajectory analysis.

The Monocle 3 (version 1.0.0; https://github.com/cole-trapnell-lab/monocle3) package was used to infer the developmental trajectory of CD8^+^ T cells. Cell clustering was calculated by the cluster_cell function using UMAP information and a resolution setting of 1 × 10^–4^ based on the precomputed dimension reduction, project, and embeddings in Figure 5A. The trajectory outline was generated by the learn_graph function using default settings. The naive CD8^+^ T cells were specified as the starting point of the trajectory by the order_cell function.

### Intercellular communication analysis.

We selected tissue cells and immune cells in LGs to calculate the variety and intensity of interaction signaling. The normalized gene expression was input to the pipeline. Secreted signaling in cellchatDB was selected to screen the specific signaling analyzed by CellChat (version 1.1.0; https://github.com/sqjin/CellChat) using default settings.

### scTCR-seq data analysis.

scRepertoire (version 1.0.2; https://github.com/ncborcherding/scRepertoire) was used to perform clonal analysis. Intercellular interaction was analyzed using scTCR-seq data. The input data used the filtered contig annotation were obtained from CellRanger. Based on hashtag labeling information, PBMCs and gland cells were separated into different groups. We used the combindTCR function to filter cells with at least one NA value and retain T-AB for further analysis. We used the compareClonotypes function to display the shared clones between samples or different clusters, visualized by the ggalluvial package. We used the clonalHomeostasis, clonalOverlap, and clonalDiversity function to determine diversity and analyze TCR information of different samples. To integrate with Seurat objects, the CombineExpression function was used for analysis (filterNA parameter was TRUE) and group cells by disease and tissue information. Amino acid information was selected for all cloneCall parameters in the above functions. We used the expression2list function to analyze the TCR information for specific clusters. We used the exportable = T parameters to obtain a table for further analysis and visualize in R using the ggplot package.

### scBCR-seq data analysis.

The Immcantation toolbox (version 4.0.0; https://github.com/immcantation/immcantation-lab) was used to process the BCR-seq data. The filtered_contig.fasta and filtered_contig_annotations.csv of each sample from CellRanger were aligned to Ig genes by the Change-O package (http://clip.med.yale.edu/changeo) using IgBLAST and IMGT germline sequence databases with default parameter values unless otherwise noted. R package alakazam (version 1.2.0) was performed for further analysis. We used the estimateAbundance function to calculate BCR abundance. alphaDiversity function was used to analyze BCR alpha diversity.

### Flow cytometry.

For surface marker staining, cells were blocked with serum obtained from C57BL/6 mice at 4°C for 15 minutes, and then stained with fluorochrome-conjugated antibodies at 4°C for 20 minutes. The antibodies against CD45 (clone HI30), CD3 (clone OKT3), CD56 (clone 5.1H11), CD8α (HIT8α, clone RPA-T8), TCRVα7.2 (clone 3C10), CD45RA (clone HI100), HLA-DR (clone L243), CD4 (clone OKT4), CD69 (clone FN5D), γδTCR (clone B1), CCR7 (clone G043H7), and CD103 (clone Ber-ACT8) were purchased from BioLegend; the antibodies against CD45 (clone 2D1), CD122 (clone Mik-β3), and CXCR6 (clone 13B1E5) were purchased from BD Biosciences. For intracellular granzyme staining, cells were fixed and permeabilized with a Cytofix/Cytoperm Fixation/Permeabilization Kit (BD Biosciences) after surface marker staining, and then stained for GZMA (clone CB9, BioLegend), GZMB (clone QA16A02, BioLegend), and GZMK (clone GM26E7, BioLegend). Flow cytometric analysis was performed on a Cytek Aurora, and data were analyzed with FlowJo software (BD Biosciences).

### Immunohistochemistry staining.

Multiplex immunohistochemistry staining was performed following the protocol of the Opal Polaris 7-Color Manual IHC Kit (Akoya Bioscience, NEL861001KT). The antibody against CD8 (catalog 70306) was purchased from Cell Signaling Technology, and the antibodies against CD20 (catalog ab78237), CD103 (catalog ab224202), Ki67 (catalog ab16667), IgG (catalog ab109489), IgA (catalog ab97215), and CD138 (catalog ab128936) were purchased from Abcam. Slides were imaged and whole slides scanned using a Vectra Polaris Automated Quantitative Pathology Imaging System (Akoya Biosciences), and multispectral images were acquired using Phenochart software, version 1.0.12 (Akoya Biosciences) to unmix and remove autofluorescence. Cell numbers and percentage of GZMK^+^CD103^–^CD8^+^ cells were quantified using Halo software (Indica Labs). Focus area in LG biopsies was calculated by HALO software. Focus score was the number of foci per 4 mm^2^ of tissue.

### RNAscope in situ hybridization.

Paraffin-embedded LG tissue biopsies were processed for RNA in situ detection using the RNAscope Multiplex Fluorescent Reagent Kit v2 (323100, Advanced Cell Diagnostics, Inc.) according to the manufacturer’s instructions. RNAscope Probe-Hs-CXCL16-No-XMm (498001, Advanced Cell Diagnostics, Inc.) was used for in situ hybridization, and followed with anti-CD8 (catalog 70306, Cell Signaling Technology) antibody staining.

### Measurement of IL-15 levels.

Blood plasma was obtained from whole blood by 450*g* centrifugation. Detection of IL-15 concentrations in the plasma was performed with an ELISA kit for human IL-15 (BMS2106, Invitrogen).

### In vitro induction of CXCR6^+^GZMK^+^CD8^+^ T cells.

CD8^+^ T cells from PBMCs of health donors were enriched with CD8 Microbeads (Miltenyi Biotec) by magnetic-activated cell sorting (MACS). Purified CD8^+^ T cells were cultured for 48 hours in T cell medium with recombinant human IL-2 (rhIL-2; 25 ng/mL), rhIL-15 (25 ng/mL), and STAT5 inhibitor (100 μM). All cytokines were purchased from PeproTech, Inc. STAT5 inhibitor (STAT5-IN-1, 285986-31-4) was purchased from Med Chem Express.

### Statistics.

Data were analyzed using R (RStudio) and GraphPad Prism 8. Results in all figures are expressed as mean ± SD. For 2-group comparisons, a 2-tailed Student’s *t* test was used to generate *P* values. A paired Student’s *t* test was used to compare the differences between cell populations from the same patient or same healthy donor in Figure 3D and Supplemental Figure 4. Multiple sample comparisons were made using 1-way ANOVA with Dunnett’s multiple-comparison test. Pearson’s correlation was used to calculate the significance of correlations. *P* values less than 0.05 were considered significant: *P < 0.05, **P < 0.01, ***P < 0.001.

### Data availability.

The data that support the findings of this study have been deposited into the CNGB Sequence Archive (CNSA) (40) of the China National GeneBank DataBase (CNGBdb) (41) with accession number CNP0003733.

### Study approval.

The research followed the Declaration of Helsinki, and the study protocols were approved by Guangdong Provincial People’s Hospital (KY2020-334-01). Participants gave written informed consent to participate in the study before taking part in the study.

## Author contributions

TX, HXZ, XY, YL, ZXL, and CYG conceived and designed the experiments, analyzed data, and wrote the manuscript. TX, HXZ, XY, and CYG carried out the experiments. TX and YL collected clinical samples. JFM and PYL helped with flow cytometry and single-cell sequencing experiments. XL co-supervised the study and revised the manuscript. All authors read and approved the final manuscript. The order of co–first authors was determined by the time at which they started working on this project.

## Supplementary Material

Supplemental data

Supplemental table 5

## Version 1 03/07/2023

In-Press Preview

## Version 2 04/24/2023

Electronic publication
