# Supplementary material for: Single-cell profiling reveals pathogenic role and differentiation trajectory of granzyme K+CD8+ T cells in primary Sjögren’s syndrome
Source: JCI Insight. 2023 Apr 24;8(8):e167490. doi: 10.1172/jci.insight.167490 (PMC10243796; doi:10.1172/jci.insight.167490)
Supplement: Supplemental data [file jciinsight-8-167490-s102.pdf]

Supplementary Information

Single-cell profiling reveals pathogenic role and differentiation trajectory of GZMK$^+$CD8$^+$T cells in primary Sjögren's syndrome

Ting Xu#, Haoxian Zhu#, Xing You#, Jinfen Ma, Xin Li, Panyue Luo, Yang Li*, Zhexiong Lian*, Caiyue Gao*

*Corresponding authors: carolgoh@mail.ustc.edu.cn (Caiyue Gao); zxlian@gdph.org.cn (Zhexiong Lian); liyang@gdph.org.cn(Yang Li)

Supplementary materials:
Supplementary Figures 1-6
Supplementary Tables 1-4

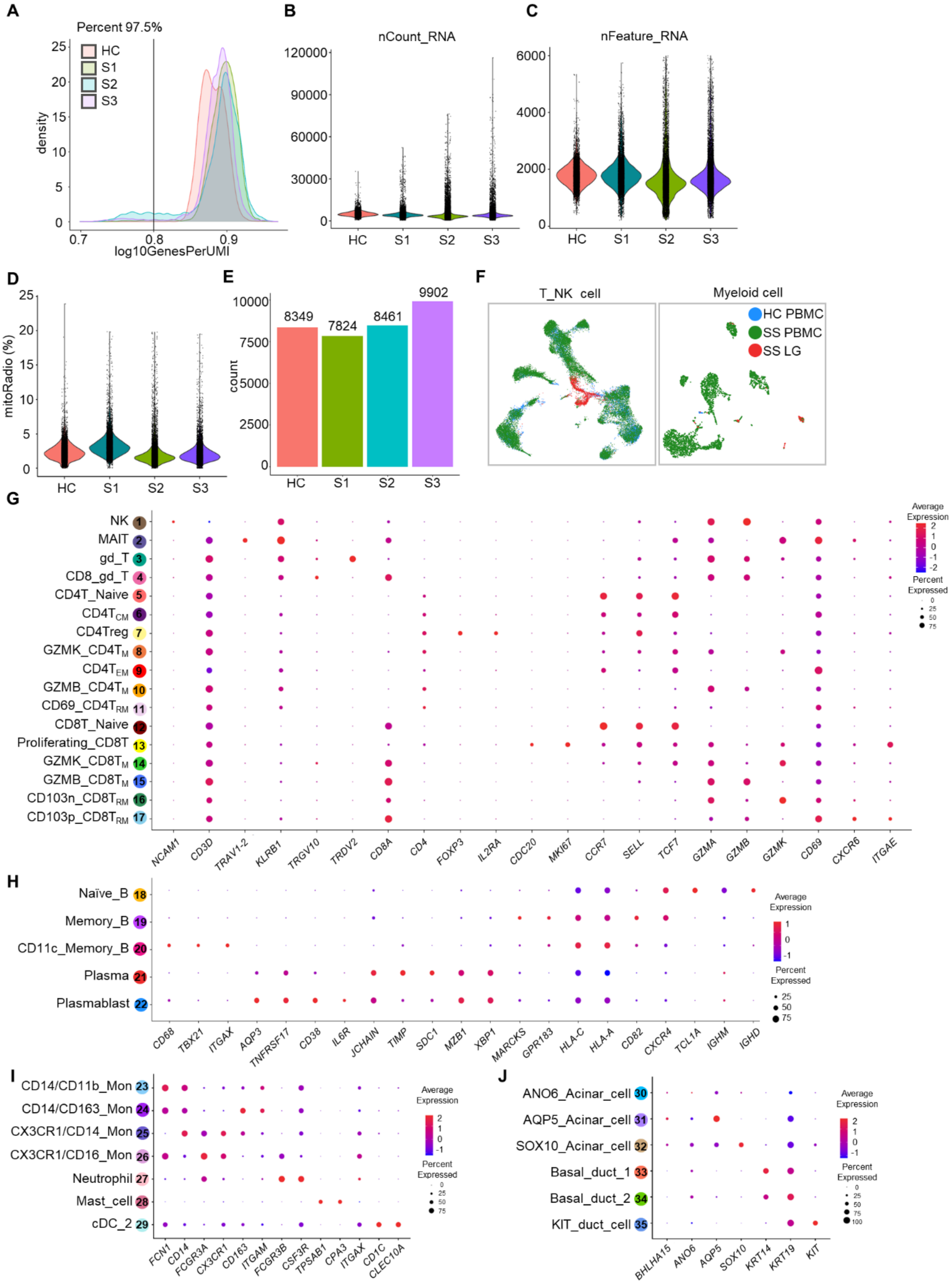

Supplementary Figure 1 Data quality and subset characteristic of scRNA-seq data of LG and PBMC in pSS patients.

(**A**) Data complexity of different samples. (**B, C**) Gene number and UMI number of different samples after quality control. (**D**) Proportion of mitochondrial gene counts in different samples after data filtering. (**E**) Total number of cells in different samples. (**F**)

UMAP plot shows the tissue distribution of T_NK cell and myeloid cell clusters. (**G-J**) Dot plot shows the marker genes of T_NK cell (**G**), B cell (**H**), myeloid cell (**I**) and tissue cell (**J**). The color of dots represents the average expression of genes and size for the percent of cells expressed the genes.

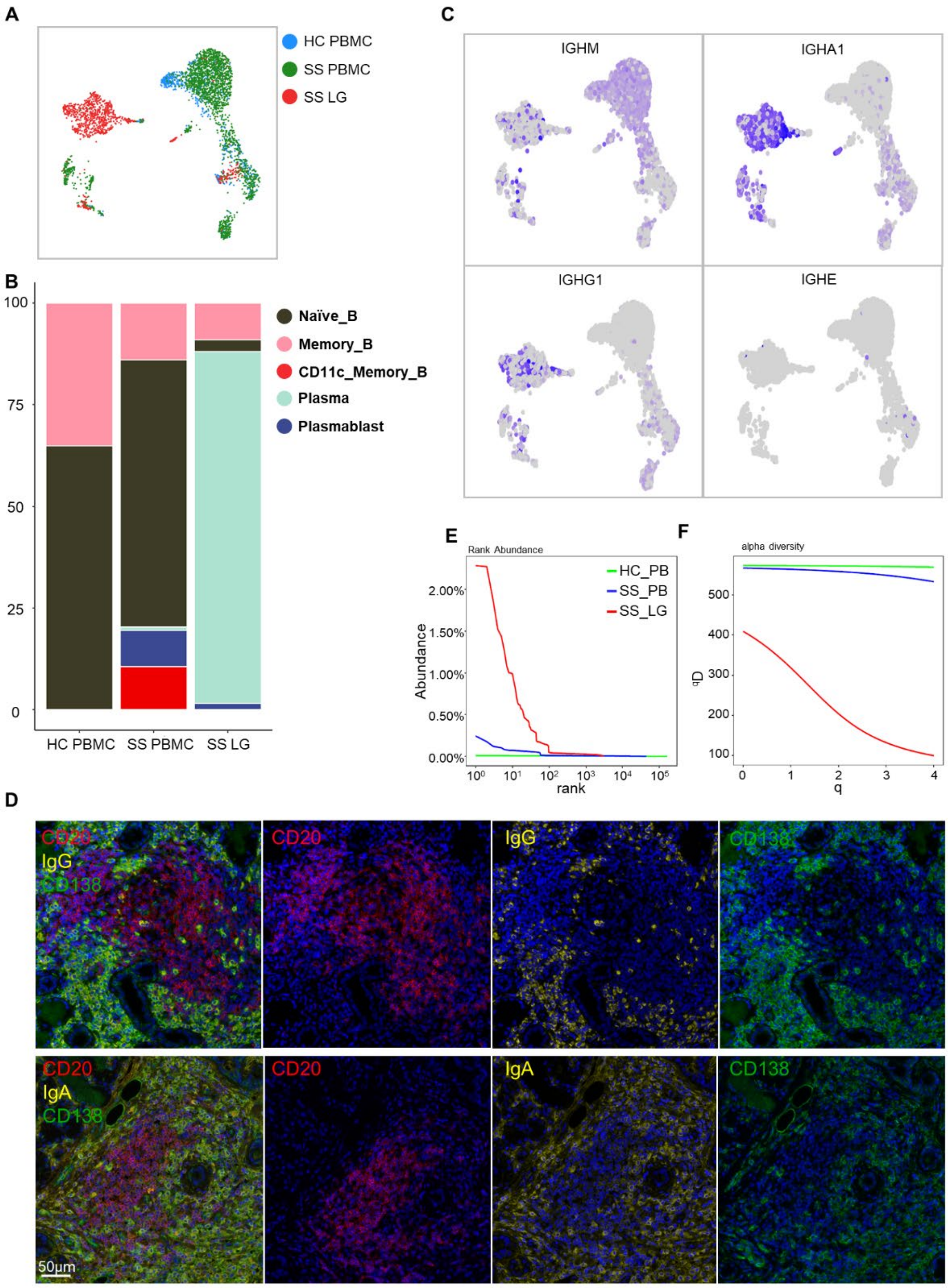

Supplementary Figure 2 Single cell transcriptional features of B cells in pSS patients.

(**A**) UMAP plot shows the tissue distribution of B cell. (**B**) Bar plot shows the proportion of five B cell subsets from HC and pSS patients. (**C**) Featureplot shows *IGHM/IGHA1/IGHG1/IGHE* expression in B cell from HC and pSS patients. (**D**) Representative multiplex immunohistochemical of CD20 (red), CD138 (green), IgG or IgA (yellow) in SS LG. Scale bar, 50μm. (**E**) Alpha diversity plot shows the BCR diversity of SS

LG and PBMC from HC and pSS patients. (**F**) Rank abundance curve shows the clone abundance of BCR in SS LG and PBMC from HC and pSS patients.

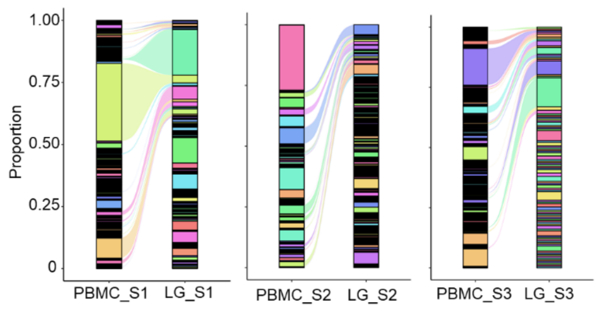

Supplementary Figure 3 Clonal expansion in CD8$^+$T cells of pSS patients.

Alluvial plot showing the share clones among LG and PBMC CD8$^+$T cells from each patient.

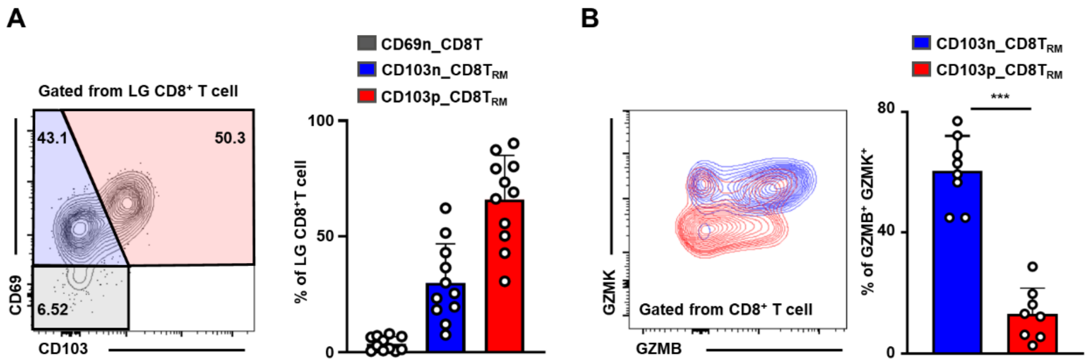

Supplementary Figure 4 Phenotype of CD8^{+}T$_{RM}$ cells in LG of pSS patients.

(**A**) Flow cytometry analysis showing the expression of CD69/CD103 in CD8+TRM cells in LG from pSS patients (n =11). (**B**) Flow cytometry analysis showing higher proportion of GZMB^{+}GZMK^{+} in CD103^{-}CD8^{+}T$_{RM}$ (n =8). Data are presented as mean ± SD. ***P < 0.001. 2-tailed paired t test (**B**) were used to compare variables.

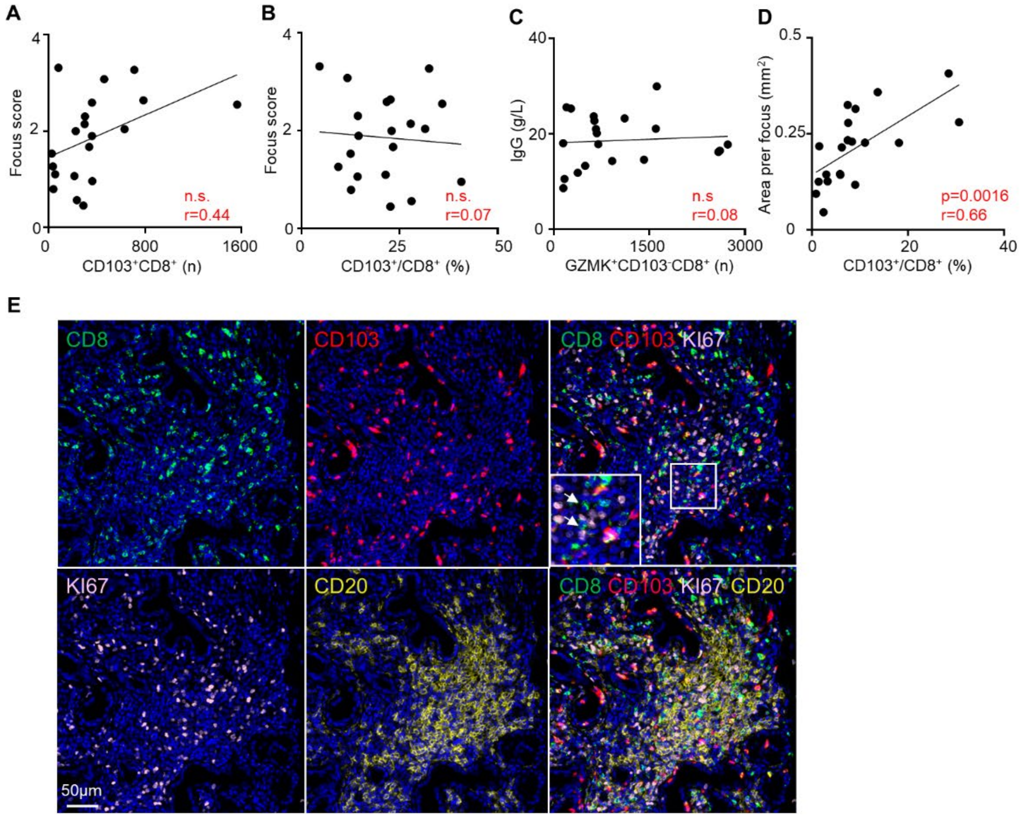

Supplementary Figure 5 Pathogenic role of GZMK+CD8+T cell in pSS.

(**A, B**) Relationship between LG focus score and $CD103^+CD8^+$ cell number or frequency (n =20). (**C**) Relationship between serum IgG concentration and $GZMK^+CD103^-CD8^+$ cell number (n =20). (**D**) Correlation between frequency of $CD103^+CD8^+$ cell and area per focus (n =20). (**E**) Representative multiplex immunohistochemical of CD20 (yellow), CD103 (red), CD8 (green) and Ki67 (pink) in pSS LG biopsy. Scale bar, 50μm. The experiment was performed 2 times. Pearson's correlation (**A-D**) was used to compare variables.

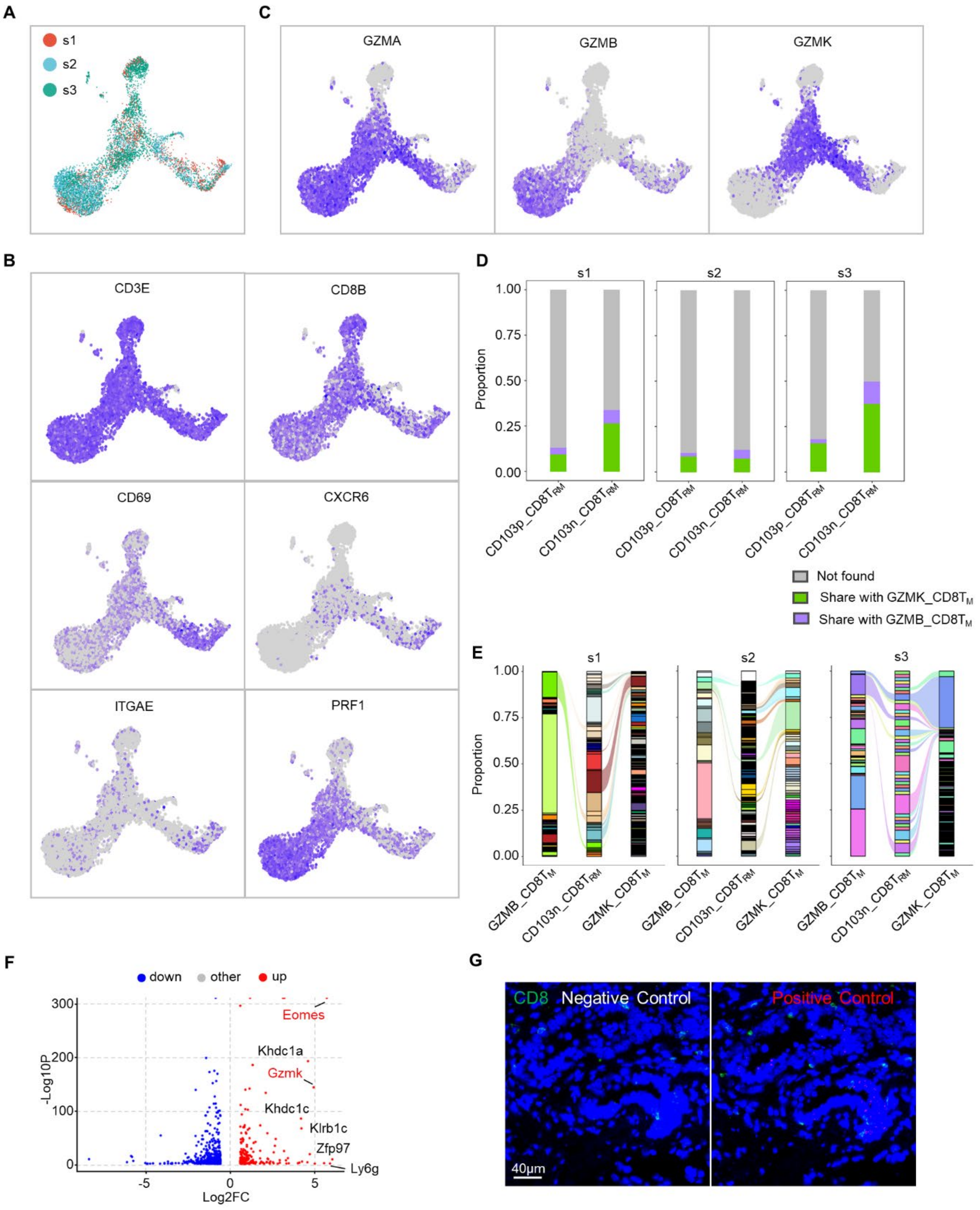

Supplementary Figure 6 GZMK+CXCR6+CD8+T cell is TRM precursor.

(**A**) UMAP plot shows the patients distribution of CD8$^+$T cells in LG and PBMC from pSS patients. (**B**) Featureplot shows the expression of *CD3E /CD8B /CD69 /CXCR6 /ITGAE /PRF1* in CD8$^+$T cell of pSS patients. (**C**) Featureplot shows the expression of *GZMA /GZMB /GZMK* in CD8$^+$T cell of pSS patients. (**D**) Percent bar graph shows the variety

proportion of shared clone with $CD103^{+}T_{RM}$ or $CD103^{-}T_{RM}$ from each patient. Grey represents variety proportion of the clones not found in GZMK_CD8T_M or GZMB_CD8T_M, while green represents variety proportion of the clones shared with GZMK_CD8T_M and purple for variety proportion of the clones shared with GZMB_CD8T_M. (**E**) Alluvial plot shows the shared clones with $CD103^{-}CD8^{+}T_{RM}$ in GZMK_CD8T_M or GZMB_CD8T_M. (**F**) Volcano plot shows differentially expressed genes between and Eomes-overexpressing and control mouse CD8+T cell. (**G**) Representative images of negative and positive control for RNAscope in situ hybridization.

Supplementary Table 1 Demographic characteristics for scRNA-seq individuals

Characteristic	pSS (n=3)	HC (n=4)
Sex, female (%)	3 (100%)	4(100%)
Age, mean (range), years	59.0(48.0-66.0)	48.8(40.0-57.0)
Disease period at sampling, median (range), years	3.0(1.0-6.0)	NA
Right eye tear flow, median (range), mm in 5 min	3.0(2.0-4.0)	NA
Left eye tear flow, median (range), mm in 5 min	2.0(2.0-5.0)	NA
Positive for ANA, n (%)	3 (100%)	NA
Positive for anti-Ro52 antibodies, n (%)	2(67%)	NA
Positive for anti-Ro60 antibodies, n (%)	2(67%)	NA
Positive for anti-La antibodies, n (%)	0(0%)	NA
IgG, median (range), g/L	12.7(11.9-30.0)	NA
IgA, median (range), g/L	2.3(1.5-8.7)	NA
IgM, median (range), g/L	1.5(1.4-2.1)	NA
CRP, median (range), mg/L	1.7(0.3-45.5)	NA
ESR, median (range), mm/H	42.0(15.0-111.0)	NA
Biopsy focus score (number of lymphocytic foci/4 mm2), median (range)	1.0(0.6-2.3)	NA
ESSDAI score, median (range)	3.0(2.0-7.0)	NA
ESSPRI score, median (range)	3.0(2.0-5.0)	NA

pSS: primary Sjogren’s disease; HC: Healthy control; NA: Not applicable; ANA: Antinuclear antibody; CRP: C-reactive protein; ESR: Erythrocyte sedimentation rate; ESSPRI: European League Against Rheumatism (EULAR) Sjögren's Syndrome Patient Reported Index; ESSDAI: EULAR Sjögren's Syndrome Disease Activity Index.

Supplementary Table 2 Demographic characteristics for recruited individuals

Characteristic	pSS (n=55)	HC (n=29)
Sex, female (%)	47 (85%)	24 (83%)
Age, mean (SD), years	49.3(15.6)	44.9(11.6)
Disease period at sampling, median (25th, 75th quartiles), years	4.0(1.0,7.0)	NA
Right eye tear flow, median (25th, 75th quartiles), mm in 5 min	5.0(3.0,6.0)	NA
Left eye tear flow, median (25th, 75th quartiles), mm in 5 min	5.0(3.0,7.0)	NA
Positive for ANA, n (%)	52 (95%)	NA
Positive for anti-Ro52 antibodies, n (%)	44(80%)	NA
Positive for anti-Ro60 antibodies, n (%)	44(80%)	NA
Positive for anti-La antibodies, n (%)	19(35%)	NA
IgG, mean (SD), g/L	18.4(6.8)	NA
IgA, mean (SD), g/L	3.1(2.2)	NA
IgM, mean (SD), g/L	1.2(0.6)	NA
CRP, median (25th, 75th quartiles), mg/L	0.8(0.5,1.8)	NA
ESR, median (25th, 75th quartiles), mm/H	27.0(12.0,40.0)	NA
Biopsy focus score (number of lymphocytic foci/4 mm^2), median (25th, 75th quartiles)	1.0(0.4,2.0)	NA
ESSDAI score, median (25th, 75th quartiles)	4.0(2.0,6.0)	NA
ESSPRI score, mean (SD)	3.0(1.5)	NA

pSS: primary Sjogren’s disease; HC: Healthy control; NA: Not applicable; ANA: Antinuclear antibody; CRP: C-reactive protein; ESR: Erythrocyte sedimentation rate; ESSPRI: European League Against Rheumatism (EULAR) Sjögren's Syndrome Patient Reported Index; ESSDAI: EULAR Sjögren's Syndrome Disease Activity Index.

Supplementary Table 3 Reference genesets used to calculate TRM score or GSEA analysis

**REACTOME_INTERFERON_GAMMA_SIGNALING**			**CD8_Cytotoxic**		**TRM_UP**	**TRM_DOWN**
B2M	*HLA-F*	*PTPN11*	*CCL5*	*CMC1*	*CD69*	*S1PR5*
CAMK2A	*HLA-G*	*PTPN2*	*GZMK*	*APOBEC3H*	*PDCD1*	*S1PR1*
CAMK2B	*HLA-H*	*PTPN6*	*GZMA*	*CST7*	*CXCR6*	*KLF2*
CAMK2D	*ICAM1*	*SOCS1*	*GZMB*	*CX3CR1*	*ITGA1*	*CX3CR1*
CAMK2G	*IFI30*	*SOCS3*	*GZMK*	*FCRL6*	*CCR4*	*EOMES*
CD44	*IFNG*	*SP100*	*GZMH*	*TMCC3*	*CTLA4*	*TBX21*
CIITA	*IFNGR1*	*STAT1*	*GZMM*	*PLA2G16*	*CXCR3*	*SELL*
FCGR1A	*IFNGR2*	*SUMO1*	*PRF1*	*TYROBP*		*CCR7*
FCGR1B	*IRF1*	*TRIM10*	*IFNG*	*TPRG1*		
GBP1	*IRF2*	*TRIM14*	*FASLG*	*C12orf75*		
GBP2	*IRF3*	*TRIM17*	*TNFSF10*	*PLCG2*		
GBP3	*IRF4*	*TRIM2*	*TNFA*	*PLEK*		
GBP4	*IRF5*	*TRIM21*	*GNLY*	*RCAN2*		
GBP5	*IRF6*	*TRIM22*	*TRGC2*	*DKK3*		
GBP6	*IRF7*	*TRIM25*	*FGFBP2*	*ADRB2*		
GBP7	*IRF8*	*TRIM26*	*C1orf21*	*FCRL3*		
HLA-A	*IRF9*	*TRIM29*	*KLRF1*	*NKG7*		
HLA-B	*JAK1*	*TRIM3*	*FCGR3A*	*PPP2R2B*		
HLA-C	*JAK2*	*TRIM31*	*PTGDR*	*SYNGR1*		
HLA-DPA1	*MID1*	*TRIM34*	*KLRC2*	*KLRC4*		
HLA-DPB1	*MT2A*	*TRIM35*	*EOMES*	*HLA-DPB1*		
HLA-DQA1	*NCAM1*	*TRIM38*	*S1PR5*	*DAPK2*		
HLA-DQA2	*OAS1*	*TRIM45*	*CLIC3*	*F2R*		
HLA-DQB1	*OAS2*	*TRIM46*	*AOAH*	*KIR3DL2*		
HLA-DQB2	*OAS3*	*TRIM48*	*CADM1*	*B3GAT1*		
HLA-DRA	*OASL*	*TRIM5*	*TRGC1*	*CD8B*		
HLA-DRB1	*PIAS1*	*TRIM6*	*DTHD1*	*TTC16*		
HLA-DRB3	*PML*	*TRIM62*	*LILRB1*	*GALNT3*		
HLA-DRB4	*PRKCD*	*TRIM68*	*SAMD3*	*SCD5*		
HLA-DRB5	*PTAFR*	*TRIM8*	*ZNF683*	*PDGFD*		
HLA-E	*PTPN1*	*VCAM1*	*KLRD1*	*ABCB1*		
			NCR1	*MXRA7*		
			FAM49A	*CTBP2*		
			KLRG1	*CD8A*		
			CTSW	*ZEB2*		
			CD244	*SYTL2*		

Supplementary Table 4 Type 2 IFN signaling network CellChat among major cell type in LG from SS patients.

source	target	ligand	receptor	prob	pval	annotation	evidence
CD69_CD8TRM	AQP5_Acinar_cell	IFNG	IFNGR1_IFNGR2	0.002522	0	Secreted Signaling	KEGG: hsa04060
CD8_gd_T	AQP5_Acinar_cell	IFNG	IFNGR1_IFNGR2	0.001968	0	Secreted Signaling	KEGG: hsa04060
CD69_CD8TRM	Basal_Duct_1	IFNG	IFNGR1_IFNGR2	0.006664	0	Secreted Signaling	KEGG: hsa04060
CD8_gd_T	Basal_Duct_1	IFNG	IFNGR1_IFNGR2	0.005205	0	Secreted Signaling	KEGG: hsa04060
CD69_CD8TRM	Basal_Duct_2	IFNG	IFNGR1_IFNGR2	0.005752	0	Secreted Signaling	KEGG: hsa04060
CD8_gd_T	Basal_Duct_2	IFNG	IFNGR1_IFNGR2	0.004492	0	Secreted Signaling	KEGG: hsa04060
CD69_CD8TRM	CX3CR1_CD14_Mo	IFNG	IFNGR1_IFNGR2	0.018089	0	Secreted Signaling	KEGG: hsa04060
CD8_gd_T	CX3CR1_CD14_Mo	IFNG	IFNGR1_IFNGR2	0.014164	0	Secreted Signaling	KEGG: hsa04060
CD69_CD8TRM	KIT_duct_cell	IFNG	IFNGR1_IFNGR2	0.006016	0	Secreted Signaling	KEGG: hsa04060
CD8_gd_T	KIT_duct_cell	IFNG	IFNGR1_IFNGR2	0.004698	0	Secreted Signaling	KEGG: hsa04060
CD69_CD8TRM	Memory_B	IFNG	IFNGR1_IFNGR2	0.004608	0	Secreted Signaling	KEGG: hsa04060
CD8_gd_T	Memory_B	IFNG	IFNGR1_IFNGR2	0.003598	0	Secreted Signaling	KEGG: hsa04060
CD69_CD8TRM	PlasmaBlast	IFNG	IFNGR1_IFNGR2	0.002235	0	Secreted Signaling	KEGG: hsa04060
CD8_gd_T	PlasmaBlast	IFNG	IFNGR1_IFNGR2	0.001744	0	Secreted Signaling	KEGG: hsa04060
CD69_CD8TRM	SOX10_Acinar_cell	IFNG	IFNGR1_IFNGR2	0.001739	0	Secreted Signaling	KEGG: hsa04060
CD8_gd_T	SOX10_Acinar_cell	IFNG	IFNGR1_IFNGR2	0.001357	0	Secreted Signaling	KEGG: hsa04060
